# Validity and reliability testing of two instruments to measure breast cancer patients' concerns and information needs relating to radiation therapy

**DOI:** 10.1186/1748-717X-2-43

**Published:** 2007-11-25

**Authors:** Georgia KB Halkett, Linda J Kristjanson

**Affiliations:** 1 Western Australian Centre for Cancer and Palliative Care, Curtin University of Technology, Perth, Australia

## Abstract

**Background:**

It is difficult to determine the most effective approach to patient education or tailor education interventions for patients in radiotherapy without tools that assess patients' specific radiation therapy information needs and concerns. Therefore, the aim of this study was to develop psychometrically sound tools to adequately determine the concerns and information needs of cancer patients during radiation therapy.

**Patients and Methods:**

Two tools were developed to (1) determine patients concerns about radiation therapy (RT Concerns Scale) and (2) ascertain patient's information needs at different time point during their radiation therapy (RT Information Needs Scale). Tools were based on previous research by the authors, published literature on breast cancer and radiation therapy and information behaviour research. Thirty-one breast cancer patients completed the questionnaire on one occasion and thirty participants completed the questionnaire on a second occasion to facilitate test-retest reliability. One participant's responses were removed from the analysis. Results were analysed for content validity, internal consistency and stability over time.

**Results:**

Both tools demonstrated high internal consistency and adequate stability over time. The nine items in the RT Concerns Scale were retained because they met all pre-set psychometric criteria. Two items were deleted from the RT Information Needs Scale because they did not meet content validity criteria and did not achieve pre-specified criteria for internal consistency. This tool now contains 22 items.

**Conclusion:**

This paper provides preliminary data suggesting that the two tools presented are reliable and valid and would be suitable for use in trials or in the clinical setting.

## Background

Radiation therapy is commonly used in combination with surgery to treat early breast cancer. It is recommended that 83% of all breast cancer patients receive radiation therapy [1]. Previous research states that the general public is poorly educated about radiation therapy and many patients feel nervous about receiving radiation therapy prior to commencing treatment [2-5]. Because so many people diagnosed with breast cancer are suitable for radiation therapy, it is essential that health professionals understand their information needs and are able to address these needs. Appropriate information provision is likely to reduce patients' fears about radiation therapy and inclinations to decline treatment and assist patients to feel confident and comfortable about receiving radiation therapy.

A number of tools to measure information needs have been developed and tested for general use when determining cancer patient's information needs [6-8]. Considerable work has also been undertaken to improve provision of information by developing and redeveloping information booklets, videos and internet resources and by recommending improvements to the way that we communicate with patients and provide verbal information. Research has also been conducted to specifically test radiation therapy information interventions and improve the information that is routinely provided to radiation therapy patients [9-11]. Notwithstanding this work, no valid and reliable tools exist to specifically determine patient's information needs at different time points during radiation therapy. One study developed a patient experience questionnaire and included questions about four types of information: *"how treatment kills cancer; possible side effects, when to expect them, and how long they would last; how to deal with treatment side effects; and when tests and physical examinations would be performed" *[[12], p. 1606]. This questionnaire was not tested for reliability or validity and was designed for all radiation therapy patients rather than for breast cancer patients specifically. Haggmark et al. [13] developed a questionnaire with the following seven specific radiation therapy satisfaction questions: *"Why receiving radiation therapy; how radiation therapy is carried out, the radiation therapy, tailor made for you; preparation before radiation therapy; implications of the visit at the simulator unit; possible side-effects of the radiation therapy and the total information given concerning the coming radiation therapy." *Each item was rated using a Visual Analogue Scale, purporting to measure patient satisfaction with information, rather than information needs or fulfillment of information needs. Reliability and validity of the questionnaire were not assessed. Several other studies on radiation therapy information provision have used generalised information scales such as the Information Satisfaction Questionnaire [14] and the Toronto Informational Needs Questionnaire-Breast Cancer [15]. Although these questionnaires provide an understanding of general information needs, the validity of these scales in reporting radiation therapy information needs is uncertain because the items do not focus specifically on patient's information needs during radiation therapy.

Without a reliable and valid tool to measure patient's information needs related to radiation therapy, it is difficult to determine the most effective approach to patient education or tailor education interventions for specific patients. Therefore, the aim of this study was to develop psychometrically sound tools to adequately determine the information needs of cancer patients during radiation therapy.

### Theoretical framework

The overarching theoretical framework used to guide this study was the 'Information Behaviour Model' proposed by Wilson [16]. This framework describes how people behave when they are provided with information and considers what people do when they need to access information. Wilson suggests that information seeking behaviours occur when people perceive that they are in need of information [16]. Patients may seek information through formal or informal information sources. Part of the information seeking behaviour used may also involve efforts to obtain information from other people, such as health professionals, family and friends. Wilson's 1996 information behaviour model suggests that information behaviour is dependent on the context of the person's information need, what activates them to gain information, how they are coping with what is happening, intervening variables (psychological, demographic, role-related or interpersonal, environmental, source characteristics), risks and rewards involved in seeking the information and self efficacy [16]. People may be active or passive information seekers. Regardless of whether people are active or passive information seekers, when they enter the hospital doors as a patient they are often routinely provided with set information about their disease and recommended treatment. A tool to determine patient's information needs would assist health professionals to provide information that meets their needs and information seeking behaviour.

Development of the two scales was informed by Wilson's research on information behaviour [16], which proposes that patients seek information when they are in need of specific information. Patients are often routinely provided with large amounts of information; however, not all of this information meets their needs and may be discarded if patients do not see the content of the information as meeting their individual needs. Patient's information seeking behaviour is dependent on the issues that concern them and as a consequence of a "need perceived by an information user" [[16], p. 3]. The first scale, the 'RT Concerns Scale', aims to measure patients concerns about radiation therapy. The second scale, the 'RT Information Needs Scale' measures the specific information needs patients may have in relation to radiation therapy. Individual items included in these scales were based on previous studies on patient's experiences of radiation therapy and the investigators' qualitative research on patient's information needs during radiation therapy.

## Methods

Ethical approval was obtained from Curtin University of Technology and Sir Charles Gairdner Hospital prior to commencement of data collection.

### Procedure for instrument development

Literature related to radiation therapy information needs of breast cancer patients was reviewed to determine the areas that had previously been addressed in data collection instruments and identify areas where improvements were required to develop a more complete understanding of these patients information needs over time. Possible scale items identified from the two studies [12,13] which appeared relevant were:

• How treatment kills cancer

• Possible side effects, when to expect them, and how long they would last

• How to deal with treatment side effects

• When tests and physical examinations would be performed

• Why receiving radiation therapy

• How radiation therapy is carried out

• The radiation therapy, tailor made for you

• Preparation before radiation therapy

• Implications of the visit at the simulator unit.

Qualitative research studies were also reviewed to determine whether additional information needs had been identified in these studies. Long [3] conducted interviews with 20 radiation therapy patients to identify what the experience of receiving radiation therapy was like and identified that the first day of treatment was difficult for patients and linked information provision to control. Gamble [5] similarly conducted a study to gain understanding of the experience of receiving radiation therapy and identified through interviews with 15 radiation therapy patients that patients required information about the cost of treatment and what would happen during radiation therapy.

Having reviewed the literature, the researchers conducted semi-structured interviews with 34 breast cancer patients and 14 health professionals to further determine the information needs that breast cancer patients had before, during and after their radiation therapy treatment. Patients were recruited from Sir Charles Gairdner Hospital and through using local media advertisements. Patients were interviewed at four time points: following their initial appointment with their radiation oncologist, after their planning appointment, following the first day of treatment and after treatment completion. The sample of health professionals included: two general practitioners; one medical oncologist, two breast surgeons, two radiation oncologists, two breast care nurses, two radiation therapy nurses and three radiation therapists. The constant comparative method was used to analyse the data. The analysis revealed that patient's information needs were specific to radiation therapy and demonstrated that some of these needs were not being adequately met. Therefore, it was clear that previous questionnaires that measured general information needs and specific breast cancer information needs were not appropriate if we wanted to gain an understanding of patient's information needs at different time points during their radiation therapy. Patients described all of the issues identified in the review of the literature above as well as a number of additional items relating specifically to the experience of receiving radiation therapy. Based upon these sources, two specific tools were then developed: the 'RT Concerns Scale' and the 'RT Information Needs Scale'. Items for these scales were based on the interview data, considering the overarching Information Behaviour Model and referring back to the literature in the area.

Content validity of the items was determined by asking a sample of six female experts (the panel of experts included one radiation therapist, two registered nurses, two females from the general population and one breast care nurse) to read each item and respond to four questions:

• Is the questionnaire clear?

• Do the items included match the topic (radiation therapy information needs)?

• Are any of the items redundant/repetitive?

• Do the items in the questionnaire also seem to be asking about the same general topic?

These questions were adapted from a template developed for assessing content validity of scale items by Mastaglia et al. [17].

Experts agreed that all items fit were appropriate. A few minor suggestions for wording improvements were made; however, no items were considered to be redundant.

### Methods for testing and refining the tools

We then conducted a survey to assess the psychometric properties of the tools. Breast cancer patients who had completed radiation therapy were recruited via media and radiation oncologists based at Sir Charles Gairdner Hospital. Each survey pack included a letter to the participant, information sheet about the study, written informed consent form demographic data collection form, and the radiation therapy information needs questionnaire. This questionnaire contained the two scales that had been developed (RT Concerns Scale and RT Information Needs Scale), categorical questions to determine patients preferences for different sources of information, qualitative questions about patients information needs and preferences, the Hospital Anxiety and Depression Scale [18], the Patient Enablement Instrument [19] and the Cancer Behaviour Inventory [20]. The results of the other scales used in this questionnaire will be reported elsewhere. The RT Information Needs scale also asked patients whether they felt that each of these information needs had been met, partially met or unmet. To allow assessment of the stability of the instrument over time, once the first questionnaire containing the two scales was returned, the second questionnaire was mailed to participants who were asked to complete this questionnaire within ten days of the initial questionnaire.

#### Approaches to Reliability Assessment

##### Internal Consistency

Internal consistency reliability estimates were calculated using Cronbach's alpha coefficient. A coefficient of greater than or equal to 0.70 was the preset as the acceptable criterion for reliability for the scale [21]. The criterion set for item-to-total correlations required that at least 50% of retained item scores correlated with total scores in the range 0.30 to 0.70 [22]. Fifty percent of inter-item correlations also had to fall within the range 0.30 to 0.70 [21].

##### Stability

The time interval between completion of the two copies of the questionnaire was to be ten days. This time interval would prevent participants from memorizing their responses. This time interval was also appropriate because patients had all completed radiation therapy and therefore their opinions about this treatment and the information they received were unlikely to change during this time period. It is essential when testing the stability of an instrument over time that the researchers select a time interval that does not allow for changes in the phenomenon during this time interval [23]. We computed correlations between T1 and T2 data to assess the stability of the instrument over time, using the Intraclass Correlation Coefficient (ICC) and 95% limits of agreement. Intraclass coefficients are used to determine whether the responses in the two time points are related and 95% limits of agreement were used to determine whether the responses were in agreement with each other [24]. Nunnally and Bernstein [21] suggest that an intraclass correlation of at least 0.70 may be considered to be satisfactory in such an assessment. Limits of agreement were included in the analysis as Bland and Altman [24] argued that 95% limits of agreement provide a more comprehensive understanding of whether the data collected at two time points is in agreement and within a range that the responses are most likely to fall. Intraclass coefficients were also calculated for the met, partially met and unmet scale included with the RT Information Needs Scale.

## Results

### Sample

Thirty-six questionnaires were sent to consenting participants at Time 1 and 31 were received (response rate = 86%). Of these 31 participants, thirty completed the questionnaires at Time 2. All participants were female. The data from one participant was removed from the analysis because she contacted the researcher and explained that she felt that she had completed the first questionnaire incorrectly.

The median age was 55.2 with a standard deviation of 9.6. The minimum age was 33 and the maximum age was 74. All participants were within 18 months of completing their radiation therapy at the time of completing these questionnaires. A summary of other relevant demographic variables is provided in Table 1.

**Table 1:** Frequency distribution of participants according to demographics and treatment related variables.

		Frequency
Country of birth	Australia	20
	England	4
	Other	6

Marriage status	Never Married	4
	Widowed	1
	Divorced/Separated	5
	Married/Defacto	20

Highest Education	Primary School	1
	Some High School	9
	Year 11 or equiv	6
	Year 12	4
	TAFE	4
	University degree	5
	Post grad degree	1

Employment	Full-time employed	7
	Part-time employed	6
	Unemployed	2
	Part-time self-employed	1
	Homemaker	4
	Full time student	1
	Part time student	1
	Retired	8

Surgery received	Breast conserving surgery	24
	Mastectomy	5
	Breast reconstruction	1

Received Chemotherapy		13

### Preliminary Internal Consistency Assessment

For the nine-item 'RT Concerns Scale', a Cronbach's alpha coefficient of 0.91 was obtained at Time 1 and a coefficient of 0.94 was obtained at Time 2. These estimates are well beyond the preset criterion of greater than 0.70 [21]. The mean inter-item correlation for the total scale was 0.52 (SD = 0.15) with a range of 0.17 to 0.82 at Time 1. For each item at least five of the eight inter-item correlations were between 0.30 and 0.70. In total, 59 of the possible 72 inter-item correlations (82%) achieved inter-item correlations between 0.30 and 0.70. This result exceeds the pre-specified criteria of over 50% inter-item correlation between 0.30 and 0.70 [21]. Items 8 (impact of treatment on life) and 9 (impact of treatment on future) had an inter-item correlation of 0.82 suggesting redundancy. However, these two items were retained for further testing to determine if results were similar with a larger sample size.

Four of the nine items achieved item-to-total correlations of between 0.30 and 0.70. This just meets the criterion set for item-to-total statistics, which suggests that at least 50% of retained item have scores in the range 0.30 to 0.70 for scales with less than 10 items [22]. The following five items achieved item-to-total correlation ranging from 0.73 to 0.79: 'What would happen during treatment'; 'The possibility of experiencing pain as a result of treatment'; 'Getting the information you required'; 'Impact of treatment on life'; and 'Impact of treatment on future'. These items were retained because the values did not indicate high levels of redundancy and given the pilot stage of the research warrant further testing.

In the case of the 24-item 'RT Information Needs Scale', a Cronbach's alpha coefficient of 0.86 was obtained at Time 1 and a coefficient of 0.94 was obtained at Time 2. This estimate was well beyond the preset criterion of greater than 0.70 [21]. The mean inter-item for the total scale was 0.24 (SD = 0.24) with a range of -0.40 to 0.90 for Time 1. Thirty-nine percent (213/552) of the items were within 0.30 and 0.70 for inter-item correlations which is below the pre-specified criteria of over 50% inter-item correlation between 0.30 and 0.70 [21]. Item 3 'Combining chemotherapy and radiation therapy' consistently did not fit with the other questions, because not all women received chemotherapy so it was removed from the scale prior to further testing. Item 5, which asks patients about CT was also removed because it did not achieve suitable inter-item correlations with the other items and participants often reported that they did not understand the question. The scale therefore became a 22-item scale. After removing these items, a Cronbach's alpha coefficient of 0.84 was obtained, still meeting the preset criterion and containing items considered to be more relevant and suitable for the majority of participants.

Eighteen out of twenty- two (82.%) items had item-to-total correlations between the pre-set criterion of 0.30 and 0.70. This is well beyond the criterion of 50% of items having item-to-total statistics between 0.30 and 0.70. The following items recorded item-to-total correlations below 0.30: 'What will happen after treatment is finished' (0.28), 'Why I need to receive radiation therapy' (0.26), and 'Whether I can keep working during treatment' (0.06). The item 'The radiation oncologist who is treating me' recorded an item-to-total statistic of 0.71.

Within this 22-item scale four subscales were developed to reflect patient's information needs at different time points during their radiation therapy. All subscales met the criterion for inter-item correlation and item-to-total correlations and had acceptable Cronbach's alpha as demonstrated in Table 2.

**Table 2:** Subscale analysis of RT Information Needs Scale

**Subscales and items**	**Cronbach's Alpha**	**Inter-Item Correlation**	**Item-to-Total Correlations**
**Initial information about radiation therapy** Q1 Why I need to receive radiation therapy Q2 What radiation therapy will involve Q7 The radiation oncologist who is treating me	0.75	All item correlations between 0.57 and 0.73	All items between 0.60 and 0.70.

**Information relating to planning treatment** Q4 What happens during the planning appointment Q6 How my treatment is planned Q9 How much of my breast will be treated Q11 What happens on the first day	0.76	10/12 items between 0.3 and 0.70	All items between 0.45 and 0.74.

**Information relating to first day of treatment** Q8 The roles of different staff in the department Q10 The treatment machine Q12 Why there are cameras and computers in the treatment room and what they are used for Q13 What the x-rays that are taken on the treatment machine are used for Q20 Who can provide me with information Q14 What side effects I may experience Q15 Whether the radiation will effect my heart Q16 Whether my lungs will be damaged by treatment Q17 Other people's experiences of receiving treatment Q18 How to take care of my skin	0.79	42/72 (58%) item correlations between 0.30 and 0.70.	All items between 0.30 and 0.70.

**Effect treatment will have on day to day living during treatment** Q19 Whether I can keep working during treatment Q21 What will happen after treatment is finished Q22 The cost of treatment Q23 Transport that is available Q24 Parking	0.73	9/12 (75%) item correlations between 0.30 and 0.70.	All items between 0.40 and 0.70.

### Stability over time

Stability over time was assessed using Time 1 and Time 2 data from twenty nine subjects. Interclass Coefficients and 95% limits of agreement were calculated for both scales.

For the 'RT Concerns Scale', the mean ICC was 0.60 (SD = 0.097), minimum 0.44 and maximum 0.73, with all ICCs being significant. The recommendation is that a correlation of at least 0.70 is achieved, this was achieved in 2 out 9 items. Six items items recorded ICC's between 0.5 and 0.69 (suggesting moderate correlation) and 1 item had an ICC of 0.44. The 95% limits of agreement varied for each item with the mean difference being 4.42. The most variation was seen for 'getting the information you require', where the variation in the responses for participants differed by at most 5.65 (see Table 3).

**Table 3:** ICC and 95% limit of agreements for RT Concerns Scale

	Item	Valid	Mean Diff T1 & T2	SD of Diff	Lower 95% limit of agreement	Upper 95% limit of agreement	T1 mean	T1 SD	T2 mean	T2 SD	ICC	P value
Q1	Maintaining work activity during treatment	27	0.15	1.85	-3.55	3.85	3.72	2.5	3.39	2.7	0.73	0.000
Q2	What would happen during treatment	27	-0.26	2.36	-4.98	4.46	4.72	2.2	4.82	2.6	0.54	0.002
Q3	The possibility of skin reactions as a result of treatment	27	-0.41	2.14	-4.69	3.87	5.24	2.0	5.64	2.7	0.61	0.000
Q4	The possibility of tiredness as a result of treatment	27	-0.15	2.11	-4.37	4.07	5.59	2.0	5.64	2.6	0.6	0.001
Q5	The possibility of experiencing pain as a result of treatment	27	-0.15	2.13	-4.41	4.11	4.07	2.5	3.93	2.6	0.67	0.000
Q6	The treatment machines	27	-1.04	2.19	-5.42	3.34	2.59	1.7	3.61	2.7	0.5	0.002
Q7	Getting the information you required	27	-0.63	2.51	-5.65	4.39	2.93	2.3	3.5	2.3	0.44	0.009
Q8	The impact of the treatment on your life	28	-0.25	2.56	-5.37	4.87	4.87	2.9	5.18	2.6	0.58	0.001
Q9	The impact of the treatment on your health in the future	28	0.04	2.03	-4.01	4.09	5.07	2.6	4.96	2.7	0.72	0.000

Note: SD = Standard Deviation, ICC = Interclass Coefficient

For the 'RT Information Needs Scale', the mean ICC was 0.55 (SD = 0.18), minimum 0.18 and maximum 0.79 with 20/22 ICCs less than the preset level of significance (<0.05). The recommendation is that a correlation of at least 0.70 is achieved; this was achieved in 6 out of 24 items, and 7 out of 24 items had ICCs of between 0.5 and 0.69. The 95% limits of agreement varied for each item with the mean difference being 4.25. The most variation was seen for "transport that is available", where the variation in the responses for participants differed by at most 7.79 (see Table 4).

**Table 4:** ICC and 95% limits of agreement for RT Information Needs Scale

		Valid	Mean Diff T1 & T2	SD of Diff	Lower 95% limit of agreement	Upper 95% limit of agreement	T1 mean	T1 SD	T2 mean	T2 SD	ICC	P value
Q1	Why I need to receive radiation therapy	29	0.45	1.18	-1.92	2.81	8.10	1.54	7.69	2.14	0.78	0.000
Q2	What radiation therapy will involve	29	0.55	1.38	-2.20	3.31	8.10	1.12	7.62	1.97	0.6	0.000
Q4	What happens during the planning appointment	27	0.15	2.36	-4.58	4.88	6.97	2.04	6.93	1.98	0.34	0.042
Q6	How my treatment is planned	28	0.00	1.85	-3.69	3.69	7.07	2.07	6.97	2.21	0.64	0.000
Q7	The radiation oncologist who is treating me	25	0.08	1.55	-3.02	3.18	7.19	2.33	7.07	2.40	0.79	0.000
Q8	The roles of the different staff in the department	28	0.11	2.33	-4.55	4.77	6.66	2.79	6.45	2.57	0.63	0.000
Q9	How much of my breast will be treated	28	0.32	1.70	-3.08	3.72	8.17	1.69	7.72	2.23	0.60	0.000
Q10	The treatment machine	28	0.54	1.82	-3.09	4.17	6.90	2.23	6.31	2.67	0.72	0.000
Q11	What happens on the first day	27	0.11	1.74	-3.37	3.59	7.31	2.12	7.29	2.26	0.7	0.000
Q12	Why there are cameras and computers in the treatment room and what they are used for	28	0.68	2.09	-3.50	4.86	6.34	2.54	5.66	2.74	0.68	0.000
Q13	What the x-rays that are taken on the treatment machine are used for	27	0.37	2.34	-4.31	5.05	6.96	2.17	6.48	2.18	0.42	0.000
Q14	What side effects I may experience	27	0.33	1.27	-2.21	2.88	8.50	0.96	8.00	1.34	0.18	0.177
Q15	Whether the radiation will effect my heart	27	0.04	2.53	-5.03	5.11	7.64	2.44	7.24	2.47	0.391	0.022
Q16	Whether my lungs will be damaged by treatment	27	0.81	1.88	-2.95	4.58	8.21	1.60	7.07	2.55	0.49	0.002
Q17	Other people's experiences of receiving treatment	27	0.37	2.08	-3.79	4.53	6.00	2.99	5.52	2.54	0.73	0.000
Q18	How to take care of my skin	26	0.58	1.42	-2.26	3.42	8.43	0.96	7.75	1.65	0.39	0.014
Q19	Whether I can keep working during treatment	28	-0.07	2.80	-5.67	5.53	5.31	3.66	5.31	3.12	0.68	0.000
Q20	Who can provide me with information	27	0.78	2.82	-4.86	6.42	7.11	2.22	6.24	2.44	0.27	0.078
Q21	What will happen after treatment is finished	27	0.37	1.86	-3.36	4.10	7.68	1.91	7.17	2.11	0.57	0.001
Q22	The cost of treatment	26	-0.12	2.49	-5.09	4.86	5.48	3.26	5.31	3.36	0.72	0.000
Q23	Transport that is available	26	-0.35	3.72	-7.79	7.09	3.37	3.09	3.48	3.30	0.34	0.043
Q24	Parking	27	-0.04	3.55	-7.13	7.06	5.21	3.36	5.21	3.35	0.48	0.006

(Please note Q3 and Q5 are not reported here as they were deleted following Internal Consistency Assessment.)

The 'RT Information Needs Scale' also included a subsection where patients were asked to identify whether they felt that their information needs had been met, partially met or unmet. Interclass Correlation Coefficients were calculated for each item and are reported in Table 5. Eight items had high correlations, eight items had moderate correlations and six items poor correlations over time. Further testing is required to determine the accuracy of using this scale for met/unmet needs over time.

**Table 5:** ICCs for Met, Partially Met and Unmet Needs

ICC	Items
>0.8	Q4. What happens during the planning appointment

0.7 to 0.79	Q1. Why I need to receive radiation therapy Q2. What radiation therapy will involve Q8. The roles of the different staff in the department Q9. How much of my breast will be treated Q10. The treatment machine Q20. Who can provide me with information Q21. What will happen after treatment is finished

0.6–0.69	Q14. What side effects I may experience Q17. Other people's experiences of receiving treatment Q18. How to take care of my skin Q19. Whether I can keep working during treatment Q23. Transport that is available

0.5 to 0.59	Q6. How my treatment is planned Q7. The radiation oncologist who is treating me Q11. What happens on the first day

< 0.5	Q12. Why there are cameras and computers in the treatment room and what they are used for Q13. What the x-rays that are taken on the treatment machine are used for Q15. Whether the radiation will effect my heart Q16. Whether my lungs will be damaged by treatment Q22. The cost of treatment Q24. Parking

## Discussion

This study presents two radiation therapy information scales that were tested for content validity, internal consistency and test-retest reliability. Results presented demonstrate that the scales performed well in all areas of criterion assessment. However, the results of this study are limited because the sample size used in the pilot study was a small, convenience sample. Larger sample sizes are required to verify the consistency and reliability of the scales. The scales have been included in the paper in their entirety to expedite further testing of the instruments by other researchers.

These preliminary findings indicate that the two scales: 'RT Concerns Scale' and 'RT Information Needs Scale' show potential as reliable and valid tools to measure breast cancer patient's information needs relating to radiation therapy. The items selected for inclusion in the scales were based on previous literature on radiation therapy [2,12,13] and the findings of the qualitative interviews that the researchers conducted with patients and health professionals. The theoretical model of Information Seeking Behaviour by Wilson [16] informed the decision to have two separate scales: one focusing on patient's concerns about radiation therapy and one focusing on patient's specific information needs. During data analysis researchers reconsidered this theoretical model and based their decisions on whether to keep items on both the statistical results and whether the items were information needs which were likely to lead patients to seek further information. All items retained in the scales were based on the notion that patient's information seeking behaviour is based on their need for information. Identification of these needs will enable health professionals to better meet patient's information needs and ensure that they have the information that patients require available.

Two items were deleted from the RT Information Needs Scale: 'Combining chemotherapy and radiotherapy' and 'Why Computer Tomography (CT) is necessary' because these items were not identified as information needs by the majority of patients. Chemotherapy was not an issue for 16 of the patients because they were not referred for chemotherapy and therefore did not need to receive information about it. Several patients were unsure of what the term 'Computer Tomography (CT)' meant and therefore had difficultly answering the question related to this item. The deletion of these questions strengthens the scale and ensures that all items address issues that patient's consider to be information needs during their radiation therapy experience.

The qualitative phase of this study confirmed previous research by the authors [25], which suggests that patient's information needs changed over time. The 'RT Information Needs Scale' therefore focuses on patient's information needs at different time points during their radiation therapy experience and was developed using five time related subscales. As Wilson's Information Behaviour Model [16] suggests, patients are only likely to seek information about specific things when they perceive that they have a need for this information. For example, patients are less likely to have a need for information about the planning appointment if this information need has already been met and treatment has commenced. The final subscales developed for the 'RT Information Needs Scale' were: initial information about radiation therapy; information relating to planning my treatment; information relating to first day of treatment; information about the effect treatment will have on my body and life and effect treatment will have on day to day living during treatment. Examination of these subscales demonstrated that all five subscales were internally consistency. Further analysis of these subscales and items that are included is necessary to further confirm the internal consistency of these subscales for a larger sample of breast cancer patients. Additionally, it would be of value to test this scale at different time points during the patient's experience of having radiation therapy and determine whether patient's information needs are being met as they proceed from meeting their radiation oncologist until treatment completion.

Analysis of test-retest reliability using both Intraclass Correlation Coefficient and 95% of limits of agreement demonstrated that both scales had adequate stability over time and provide reliable results. Further testing with larger and more representative samples will further verify the reliability of this scale. If future studies confirm these results, it is anticipated that these tools could be used in radiation therapy departments and have potential for adaptation for use with patients with diagnoses other than breast cancer.

## Conclusion

Two new tools for determining patient's information needs relating to radiation therapy were developed and pilot tested. These tools build on previous research on patient information needs to provide a more detailed scale that can be used specifically in radiation therapy. Both tools were tested for content validity, internal consistency and stability over time. Evidence obtained provides support for the reliability and validity of the tools. Additional testing is required to confirm these initial estimates using larger samples of breast cancer patients. Communication of these results along with the developed instruments will allow other investigators to test the instrument and expedite this area of research. If future research confirms these results, subsequent studies can be undertaken to determine the radiation-specific information needs of breast cancer patients and assess whether these information needs are being met.

## Competing interests

The author(s) declare that they have no competing interests.

## Authors' contributions

GH designed the study, carried out data collection and analysis and drafted the manuscript. LK participated in the design of the study, provided advice about data analysis and reporting the results and participated in writing the manuscript. Both authors read and approved the final manuscript.
